# Household characteristics for older adults and study background from SAGE Ghana Wave 1

**DOI:** 10.3402/gha.v6i0.20096

**Published:** 2013-06-11

**Authors:** Richard B. Biritwum, George Mensah, Nadia Minicuci, Alfred E. Yawson, Nirmala Naidoo, Somnath Chatterji, Paul Kowal

**Affiliations:** 1 Department of Community Health, University of Ghana, Accra, Ghana; 2 Institute of Neuroscience, National Council Research, Padova, Italy; 3 Multi-Country Studies unit, World Health Organization, Geneva, Switzerland; 4 University of Newcastle Research Centre on Gender, Health and Ageing, Newcastle, Australia

**Keywords:** SAGE, Ghana, ageing, household characteristics

## Abstract

**Background:**

Globally, the population aged 60 years and older is projected to reach 22% by 2050. In sub-Saharan Africa, this figure is projected to exceed 8%, while in Ghana, the older adult population will reach 12% by 2050. The living arrangements and household characteristics are fundamental determinants of the health and well-being of this population, data sources about which are increasingly available.

**Methods:**

The World Health Organization's Study on global AGEing and adult health (SAGE) Wave 1 was conducted in China, Ghana, India, Russian Federation, Mexico, and South Africa between 2007 and 2010. SAGE Ghana Wave 1 was implemented in 2007/08 using face-to-face interviews in a nationally representative sample of persons aged 50-plus, along with a smaller cohort aged 18–49 years for comparison purposes. Household information included a household roster including questions about health insurance coverage for all household members, household and sociodemographic characteristics, status of the dwelling, and economic situation. Re-interviews were done in a random 10% of the sample and proxy interviews done where necessary. Verbal autopsies were conducted for deaths occurring in older adult household members in the 24 months prior to interview.

**Results:**

The total household population was 27,270 from 5,178 households. The overall household response rate was 86% and household cooperation rate was 98%. Thirty-four percent of household members were under 15 years of age while 8.3% were aged 65-plus years. Households with more than 11 members were more common in rural areas (57.2%) and in the highest income quintile (30.6%). Household members with no formal education formed 24.7% of the sample, with Northern and Upper East regions reaching more than 50%. Only 26.8% of the household members had insurance coverage. Households with hard floors ranged from 25.7% in Upper West to 97.7% in Ashanti region. Overall, 84.9% of the households had access to improved sources of drinking water, with the lowest at 29.6% in the Volta region. The overall rate of access to improved sanitation was just 14.9%. The findings show significant regional differences, with the three Northern Regions having worse education, income, and sanitation levels, compared to Southern and Central Regions of the country.

**Conclusion:**

Household characteristics and intra-household dynamics have been shown to influence health and health-seeking behaviors across a number of contexts and countries, and play a fundamental role in the well-being of older Ghanaians. SAGE Ghana is part of a multi-country study using standardized questionnaires and tested methodologies to provide household level data required to inform policy on the growing population of older adults in Ghana. With the good response rates and measures instituted to assure quality of data, this article demonstrates the high quality data and research methods of SAGE.

Globally, declining fertility and mortality rates are contributing to a more rapid increase in older populations both in relative and absolute terms. The older adult population has increased steadily since 1950 in all the regions of the world, including Ghana where the population aged 60 years and older (60-plus) in 1950 was 4%, is currently about 6% and will reach 12% by 2050 (1). The 2010 Global Burden of Disease provides evidence of the changing trends in disease patterns with significant increases in non-communicable disease conditions amongst the general population and the older population in Ghana, where a leading health risk is household air pollution (2). Consequently, health policies and health systems need information about the emerging epidemiologic transition and its determinants in older adults to inform strategies for responding to the needs of this growing segment of the population. Households comprised solely of older persons, or multigenerational households including older household members, will rely on timely responses by social, financial and health systems to ensure the continued contributions made by older persons to families and communities (3–5).

Little information exists in Ghana regarding the situation of older people. The lack of data means that ageing is poorly understood and as a result, resources are not allocated to meet the needs of the older population. The 2000 National Population Census in Ghana and 2008 Ghana Demographic and Health Survey projected estimates of around 7% for the national proportion of the population aged 60-plus years in 2010 – as compared to 7.4% calculated from the 2010 National Population Census (6–8). In all 10 Administrative regions of Ghana, the trends in the proportion of the older population since the 2000 National Population Census have been increasing, with minor regional differences (8). Older Ghanaian women continue to face challenges with respect to abuse of property rights, while older Ghanaian men without a family are often more vulnerable than women.

The issue of lack of data about the growing population of older adults has been identified by several countries, including Ghana (9). In collaboration with the World Health Organization and support from the United States National Institute on Aging, Ghana has begun to strengthen its evidence base to inform policy through the Study on global AGEing and adult health (SAGE) (10). The national policy goal is to provide a framework that is capable of transforming and improving the lives of older persons in Ghanaian society (11). The vision is to achieve the overall social, economic, and cultural re-integration of older persons into mainstream society, and to enable them to participate fully in the national development process and to promote active ageing with adequate security and dignity. All these areas form the basis for the present study.

## Materials and methods

The 2007/08 SAGE Wave 1 in Ghana built on the 2003/04 World Health Survey (WHS), referred to as SAGE Wave 0 (12). The SAGE Wave 1 included follow-up respondents taken from Wave 0, and added new respondents to increase the cohort size for future waves. SAGE Wave 1 collected household data primarily on persons aged 50-plus years, plus a smaller cohort of adults aged 18 to 49 years for comparison purposes.

### Sampling design

Ghana used a stratified, multistage cluster design to select 251 EAs. The sample was stratified by administrative region (Ashanti, Brong Ahafo, Central, Eastern, Greater Accra, Northern, Upper East, Upper West, Volta, and Western) and type of locality (urban/rural) resulting in 20 strata and is nationally representative. The Census Enumerated Areas (CEA) of the 2000 Population and Housing Census was used as the sampling frame, and updated in 2007 through household listings/enumerations prior to interview. A sample of 251 EAs was selected as the primary sampling units (PSU). The PSUs correspond to the Ghana census enumeration areas (EAs) with well-defined boundaries, identified on maps, relatively small sized clusters that facilitated manageable interviewer workload. The number of EAs to be selected from each strata was based on proportional allocation (determined by the number of EAs in each strata specified on the census frame). EAs were then selected from each stratum with probability proportional to size; the measure of size being the number of individuals aged 50-plus years in the EA.

In each selected EA, a listing of the households was conducted to classify each household into the following mutually exclusive categories:

1. SAGE Wave 0 follow-up households with one or more members aged 50-plus years;
2. New households with one or more members aged 50-plus years;
3. SAGE Wave 0 follow-up households which did not include any members aged 50-plus years, but included residents aged 18–49; and
4. New households which did not include any members aged 50-plus years, but included residents aged 18–49.

Twenty-four households were targeted from each selected EA. All SAGE Wave 0 follow-up 50-plus households were eligible for the household interview. Twenty such households were needed and if this target number was not reached, then the balance was selected using systematic sampling from the new 50-plus households. All 50-plus members of the household were eligible for the individual interview (multiple individual interviews possible in these households).

For SAGE Wave 1, target sample sizes were 5,000 respondents aged 50-plus and 1,000 respondents aged 18–49 years old; since within each PSUs 20 households with one or more individual aged 50-plus and four households with members aged 18–49 to be selected, 250 PSUs were used.

#### Geodata

**Fig. 1:**
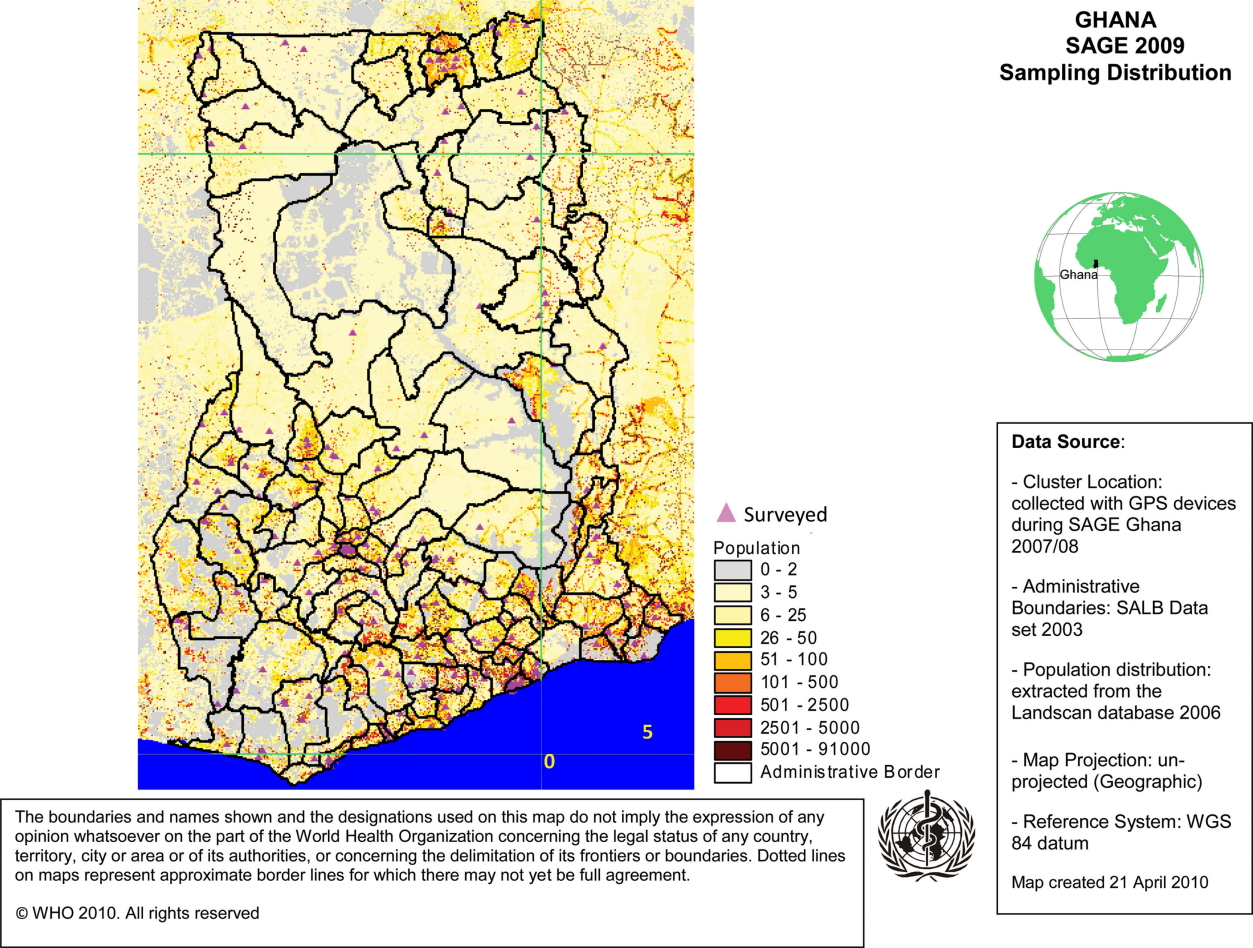
Distribution of Eas for SAGE Ghana Wave 1.

The GPS data were taken in front of respondents’ houses and a minimum of five satellites were required for readings to be accepted as accurate. It is planned that the GPS data will be used for future analysis (for example, mapping distance to health care facilities, finding respondents for the next rounds of data collection, and/or finding respondents for validation studies/sub-studies) (Fig. 1).

### Questionnaires

Six types of questionnaires were used in the SAGE study. One household informant was identified to complete the household questionnaire, and could also be selected as the individual respondent for interview using the individual questionnaire. All persons aged 50-plus in older households were invited to participate, whereas only one person was randomly selected in younger households for the individual interview. Where there was a death recorded within the 2 years prior to interview for a follow-up household, a verbal autopsy questionnaire was completed. If a selected respondent was found to be incapable of answering the individual questionnaire, a proxy respondent was identified and a proxy questionnaire completed. As part of the quality control measures, two respondents within each PSU were to be randomly selected for re-test and one for proxy validation – as part of the quality control process. In all, each PSU was to have 20 completed interviews for 50-plus respondents (household and individual), four interviews for the 18–49 year old respondents, two re-test questionnaires, one proxy validation questionnaire and verbal autopsy questionnaires where applicable.

The domains covered in the questionnaires are as follows (see also, Appendix 1):

**Household questionnaire:** 1) Contact record, sampling, GPS, re-contact information; 2) household roster including health insurance coverage of household members; 3) housing; 4) household and family support/transfers; 5) assets and household income; and 6) overall consumption and health expenditures.

**Individual questionnaire:** 1) Sociodemographic characteristics; 2) work history and benefits; 3) health state descriptions and disability; 4) anthropometrics, biomarkers, and performance tests; 5) risk factors and preventive health behaviors; 6) chronic conditions and health services coverage; 7) health care utilization and health system responsiveness; 8) social cohesion; 9) subjective well-being and quality of life; and 10) impact of caregiving.

**Verbal autopsy:** 1) Health and well-being of the deceased person(s); and 2) events leading to death.

**Re-test (Household):** 1) Household and family networks and transfers; 2) assets and household income; and 3) household expenditure.

**Re-test (Individual):** 1) Health state descriptions; 2) anthropometrics, performance tests and biomarkers; and 3) health care utilization.

**Proxy and validation:** 1) Respondent characteristics; 2) health state descriptions; 3) chronic conditions and health service coverage; and 4) health care utilization.

### Implementation

A total of 30 interviewers (experienced census officers from Ghana Statistical Service) and supervisors (principal and senior research assistants from the Department of Community Health, University of Ghana Medical School) were trained in two phases. Initially, the full survey team was trained for 10 days with support from WHO Geneva in Accra, then were divided into three teams and assigned to three survey sites (one for the three northern Regions, another for Regions in the middle belt and the other for the Regions in the south). Further training and refreshing of these interviewers was done before the start of field work. The interviews were conducted using interviewer administered questionnaire by face to face contact. Three re-contact visits were to be made before respondents were classified as unable to locate.

### Variables for analysis

Household and individual response rates were generated using by assessing adding the total completed and partially completed interviews and dividing by the number of eligible households or individuals. Information about household members (relationship to household head, age, sex, education, marital status, and health insurance coverage) and composition, dwelling characteristics (type of floors, walls and cooking stove; access to water, sanitation and cooking fuel), income and consumption were used for analyses. Improved water and sanitation provide a crucial health function. Improved water is defined as water piped into the household or yard/plot, a public standpipe, borehole, protected dug well, protected spring, rainwater collection, and bottled water. Unimproved sources include an unprotected dug well, unprotected spring, surface water and tanker truck supplies. Improved sanitation includes connection to septic system, pour-flush latrine, and private covered dry latrine. Unimproved sanitation facilities include uncovered dry latrine (without privacy), bucket latrine and no facilities (open defecation). Single and multiple generations within households were based on relationship to the household head.

Wealth or income quintiles were derived from the household ownership of durable goods, dwelling characteristics and access to services (improved water, sanitation, and cooking fuel) for a total of 21 assets (12). A two-step random effect probit model was used to generate the quintiles. An asset ladder was first generated based on the endorsement rate of the different assets. This ladder was then used to arrange household on the same scale, based on their asset ownership. The result is a continuous income score, from which quintiles are created.

### Statistical analysis

The comparison between categorical variables was performed through the Chi-square test, while for the mean comparison across groups the General Linear Model procedure was used, prior checking the homoscedasticity assumption. All analyses were performed using SAS version 9.2.

#### Weights

Data on strata sizes and household sizes for selected enumeration areas were obtained and used to calculate weights for the selected households and individual respondents. Household weights were based on the selection probability at each stage of selection, and were post-stratified by region and locality according to the 2010 household projections provided by Ghana Statistical Service (8). Post-stratification weights were based on the 2010 National Population and Housing Census. All results in this article are weighted. A link to additional information about SAGE is at www.who.int/healthinfo/sage/en.

## Results

### Response rate

The response rate in general was very high. The range for the households was between 97.1 and 100% (Table 1). For the individual, the response rates ranged between 92.1% for women and 97.0% for men. The overall response rate for households was 97.7%, slightly lower in urban (97.1%) than rural areas (98.4%; Table 1). By region, the urban strata response rate ranged from 64% for the Northern to 97% for the Central region. Among the rural strata, the lowest rate was found in the Ashanti region (71%) and highest in the Eastern region (99%).

**Table 1:** Household and individual response rates by selected background characteristics

Characteristics	HH response rate	HHS contacted	Individual* response rate	Individuals contacted
Age group				
18–49	99.2	989	95.8	792
50–59	99.7	1,535	93.4	1,695
60–69	99.8	1,059	95.4	1,162
70–79	99.9	891	95.0	981
80+	99.7	382	94.9	449
Sex				
Male	99.6	3,131	97.0	2,751
Female	99.6	2,028	92.1	2,594
Residence				
Urban	97.1	2,177	94.0	2,204
Rural	98.4	3,092	95.0	3,144
Income quintile				
Q1 (lowest)	99.9	1,027	95.7	1,031
Q2	100	1,027	94.7	1,055
Q3	99.9	1,027	94.9	1,051
Q4	100	1,027	94.8	1,097
Q5 (highest)	100	1,027	93.1	1,104
Total	97.7	5,178	93.8	5,348

* Individual here includes both individual and proxy interviews.

**Fig. 2:**
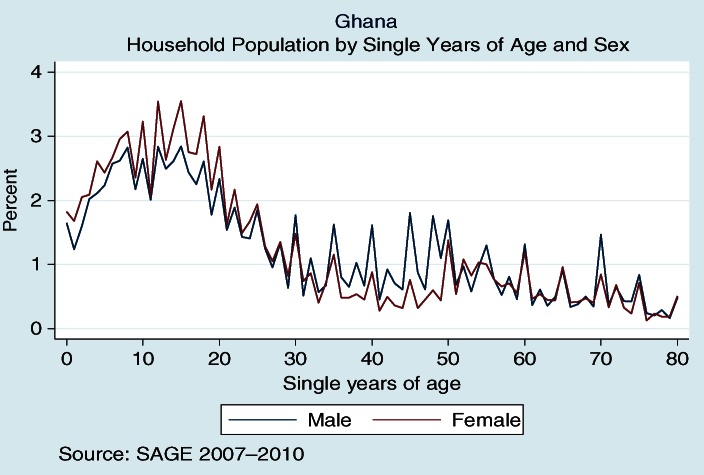
Age heaping using Myers’ blended method for household members in SAGE-Ghana.

Age is an important study variable in demography and epidemiological studies, perhaps even more important when age is a central focus, as in SAGE. Misstatement of age is one example of content error in census and surveys, with an assessment of age heaping considered a measure of data quality and consistency (13). The Myer's blended index is one such measure and examines concentration on terminal digits resulting in a score between 0 (no heaping) and 90 (all ages reported with same terminal digit). The Myers’ blended index for household members in SAGE Ghana is 11.9, which indicates that a minimum of 11.9% of the population reported ages with an incorrect final digit. The index value is very low, with some evidence of heaping on end digits 0 and 5 (Fig. 2).

#### Characteristics of the household and the household population

Table 2 shows the distribution of household population by socio-demographic/-economic characteristics and by region. The total household population was 27,270 from 5,178 households, with an average of 5.3 persons per household. The number of males was 13,196 (47.1%) with a sex ratio of 1:1.12. The percentage of females was higher in all the regions, except the three northern regions (Northern, Upper East, and Upper West). The population aged 50-plus years formed 21.0% of the overall household population with the highest in the Eastern region (26.0%) and lower in the three northern regions (lowest in Northern region [14.5%], followed by Upper West region [17.6%] and the Upper East region [19.3%]).

**Table 2:** Selected characteristics (%) of the household population and households, by region and overall

Characteristics	All	Ashanti	Brong Ahafo	Central	Eastern	Greater Accra	Northern	Upper East	Upper West	Volta	Western
Sex											
Male	47.1	46.5	46.9	46.1	45.9	45.3	51.6	51.5	53.4	45.2	46.2
Female	52.9	53.5	53.1	53.9	54.1	54.7	48.4	48.5	45.6	54.8	53.8
Age (years)											
0–14	34.0	32.6	35.0	36.7	36.3	27.3	41.8	37.1	35.7	33.0	33.4
15–49	44.9	46.1	44.2	40.5	37.7	51.8	43.6	43.7	46.8	43.4	45.2
50–65	12.7	13.4	11.6	12.7	14.8	13.7	9.4	12.1	10.3	12.5	13.5
65+	8.3	7.9	9.1	10.1	11.2	7.1	5.1	7.2	7.3	11.1	7.9
Marital status*											
Never married	41.0	42.7	41.4	39.6	34.5	48.7	35.4	31.8	39.3	37.4	44.2
Currently married	40.7	37.1	39.6	37.5	40.0	35.1	53.6	58.7	51.2	43.4	34.9
Cohabiting	2.5	2.9	2.9	2.5	3.7	1.7	3.7	0.1	0.9	1.9	2.7
Separated/divorced	6.4	7.6	6.3	8.7	9.6	6.2	1.1	1.4	1.1	5.8	9.3
Widowed	9.4	9.7	9.8	11.6	12.2	8.3	6.2	8.0	7.5	11.5	8.8
Education status **											
No formal school	24.6	18.5	23.0	23.4	18.9	13.1	52.2	53.6	44.3	21.2	18.3
Primary school	54.0	58.3	57.5	58.7	63.3	48.6	42.1	38.9	37.8	61.7	58.3
Secondary school	10.9	10.0	10.7	10.7	7.9	20.9	3.7	6.2	12.1	8.3	9.0
High school	8.3	11.0	8.0	6.1	8.8	11.7	1.5	0.6	1.6	6.5	12.7
College and higher	2.2	2.1	0.8	1.0	1.0	5.7	0.4	0.6	4.1	2.3	1.7
Residence											
Urban	51.1	59.5	45.0	44.3	40.5	89.3	29.9	18.2	24.2	31.1	43.8
Rural	48.9	40.5	55.0	55.7	59.5	10.7	70.1	81.8	75.8	68.9	56.2
Insurance coverage											
Mandatory	1.4	1.9	0.3	0.4	1.3	3.3	0.1	2.4	1.5	0.6	0.6
Voluntary	24.3	24.0	40.8	14.4	36.3	14.7	23.2	14.6	51.9	21.7	24.2
Both	1.1	1.5	0.3	0.5	0.5	0.5	0	6.1	0.2	0.9	1.9
None	73.2	72.6	58.6	84.7	61.9	81.5	76.7	76.9	46.4	76.8	73.3
Household population (n)											
	27,270	4,689	2,355	2,101	2,850	4,875	2,989	1,595	743	2,248	2,825
Household size											
1	11.0	12.8	12.5	13.7	13.7	9.6	3.1	2.8	2.9	13.2	11.6
2–5	47.2	50.8	47.6	55.9	52.0	46.9	22.3	22.1	35.8	54.1	50.2
6–10	34.8	32.7	35.1	28.3	31.2	37.3	46.4	46.9	51.9	27.4	34.7
11+	6.9	3.8	4.7	2.1	3.0	6.2	28.1	28.2	9.4	5.3	3.5
Mean household size											
	5.2	4.8	4.9	4.4	4.6	5.3	8.2	8.1	6.6	4.8	4.9
Income quintiles											
Q1	17.3	10.6	13.1	20.4	18.5	6.1	33.1	50.5	33.5	21.9	17.6
Q2	18.5	15.3	16.1	22.8	21.2	10.7	24.4	22.6	28.3	25.6	19.1
Q3	19.3	19.1	24.8	28.7	22.4	12.5	17.8	13.6	14.2	20.2	18.1
Q4	21.4	25.6	27.3	17.0	22.3	22.1	16.5	8.4	8.4	18.0	24.0
Q5	23.4	29.4	18.7	10.9	15.5	48.6	8.2	4.9	15.6	14.3	21.2
Total households (n)											
	5178	966	473	477	621	920	367	197	113	471	573

* Subjects aged (0–14) not included;

** Subjects aged (0–5) not included.

The marital status among those aged 15-plus showed 41.0% never married, 40.7% were currently married, 9.4% widowed, 6.4% were separated/divorced, and 2.5% were cohabiting. The highest percentage of those never married was in the Greater Accra region (48.7%) and lowest in the Upper West (31.8%). The three northern regions had the highest percentages of currently married respondents (Upper East region [58.7%], Northern region [53.6%] and Upper West region [51.2%]); the Western region and Greater Accra had the lowest percentage of those currently married (34.9 and 35.1%, respectively). The Eastern region had the highest percentage of widows (12.2%) and the Northern region had the lowest (6.2%). Amongst all the households members, more than half (54.0%) had only primary school education (either completed or not completed) and this percentage did not include children younger than six years. Those with no formal education constituted 24.6%, members with secondary school completed were 10.9% and those who had college education and higher were only 2.2%. The three northern regions had the highest percentages of those with no formal education (Upper East region [53.6%], Northern [52.2%] and Upper West region [44.3%]). Greater Accra had the lowest percentage of those with no formal education (13.1%), followed by the Western region (18.3%) and then the Ashanti region (18.5%). Greater Accra had the highest percentage of those with college education and higher (5.7%) and the lowest was in the Northern region (0.4%).

The majority of households (51.1%) were located in an urban setting. In the individual regions, however, only the Greater Accra and Ashanti regions had more members located in urban areas (89.3 and 59.5%, respectively), whilst the remaining eight regions had more rural dwellers.

Over 73% of all household members had no health insurance coverage, 24.3% had voluntary insurance and only 1.4% had mandatory insurance. The Central region and Greater Accra had the highest percentage of those without insurance coverage (84.7 and 81.5%, respectively), whilst the highest percentage of those with voluntary insurance was recorded in the Upper West region (51.9%), followed by the Brong Ahafo region (40.8%).

Households with two to five members were the most common size (47.2%), followed by 6–10 member households (34.8%), then the single member households (11.0%) and the least was the 11+ member households (6.9%). By region, the mean household size was highest in the Northern, Upper East and Upper West regions (8.2, 8.1, and 6.6, respectively) reflecting the 75% of households with more than six members. In all other regions, the mean size varied between 4.4 and 5.3.

About 49% of Greater Accra households belonged to the highest income quintiles, followed by a 29.4% of the Ashanti households. The three northern regions had the highest percentages in the lowest income quintile group with over half of all households in the Upper East region (50.5%) and one out of three households in the Upper West (33.5%) and Northern regions (33.1%).

Table 3 shows the characteristics of the household population by sex. Statistically significant sex differences were found for many household characteristics: the age distribution showed a higher percentage of younger males (about 67.9% aged below 29 years compared to 58.7% for females). More males lived in rural areas (52.7%) while more females were located in urban areas. More women are widowed (15.0%). For each level of education, males had higher schooling than females; males with no formal schooling accounted for 18.2% versus 30.4% for females. Although only a quarter of the household members had insurance cover, females had relatively higher cover (28.1%) compared to males (25.2%).

**Table 3:** Selected characteristics (%) of the household population, by sex

Characteristics	Male (n=12,859)	Female (n=14,411)	Total (n=27,270)	p
Age (years)				<0.0001
0–9	22.5	19.6	21.0	
10–19	28.5	23.9	26.1	
20–29	16.9	15.2	16.0	
30–39	7.3	9.2	8.3	
40–49	4.6	10.1	7.6	
50–59	8.9	8.8	8.8	
60–69	5.7	5.6	5.6	
70–79	3.8	4.9	4.4	
80+	1.8	2.6	2.2	
Residence				<0.0001
Urban	47.3	51.6	49.6	
Rural	52.7	48.4	50.4	
Marital status*				<0.0001
Never married	50.5	33.3	41.0	
Currently married	41.6	39.9	40.7	
Cohabiting	1.6	3.2	2.5	
Separated/divorced	3.8	8.6	6.4	
Widowed	2.6	15.0	9.4	
Education status**				<0.0001
No formal school	18.2	30.4	24.7	
Less than primary	35.8	33.0	34.3	
Primary school	21.2	19.2	20.1	
Secondary school	12.2	9.2	10.6	
High school	9.6	6.7	8.1	
College and higher	3.0	1.4	2.2	
Insurance coverage				<0.0001
Mandatory	1.3	1.5	1.4	
Voluntary	22.9	25.4	24.2	
Mandatory and voluntary	1.0	1.2	1.1	
None	74.8	71.9	73.2	

* Subjects aged (0–14) not included;

** Subjects aged (0–5) not included.

The distribution of some household characteristics by locality and income quintiles is shown in Table 4. Most households with five or fewer household members were located in urban areas, and 57.2% of households with 11 or more members were located in rural areas. The mean number of persons per household was 5.0 for urban and 5.6 for rural settings. Households with a respondent aged 50-plus years living alone were evenly split by location, and although households with respondent and spouse both aged 50-plus years were more prevalent in the rural areas (62.4%), no statistical association was found. Similarly, the number of generations living in the household and the location did not show any association.

**Table 4:** Selected characteristics (%) of the households, by location and income quintiles

Characteristics	Urban (n=2619)	Rural (n=2537)	p-value	Q1 (n=885)	Q2 (n=950)	Q3 (n=991)	Q4 (n=1100)	Q5 (n=1202)	p
Household size			0.012						<0.0001
1	51.5	48.5		30.6	27.5	21.8	14.1	6.0	
2–5	52.1	47.9		17.9	18.6	18.9	22.2	22.3	
6–10	50.4	49.6		13.4	15.7	19.0	23.0	28.9	
11+	42.8	57.2		11.5	18.0	20.2	19.6	30.6	
Mean household size	5.0	5.6	0.038	4.4	4.9	5.2	5.4	6.0	0.031
Living arrangements			n.s.						0.029
Single-person household, with person aged 50+	49.6	50.4		33.3	27.3	20.5	13.6	5.4	
50+ household, with only respondent and spouse	48.7	51.3		15.1	11.9	31.5	22.4	19.1	
50+ household with respondent and spouse both aged 50+	37.6	62.4		17.4	14.0	22.1	23.9	22.6	
Multigenerational household§			n.s.						<0.0001
One-generation	46.5	53.5		15.7	14.0	23.7	25.3	21.4	
Two-generation	50.5	49.5		15.8	17.6	18.3	21.5	26.8	
Skip-generation	48.9	51.1		28.6	25.4	20.0	16.7	9.2	
Three-generation	50.3	49.7		12.9	16.2	20.1	24.5	26.2	
Household head			<0.0001						<0.0001
Younger woman (aged 18–49)	64.3	35.7		15.6	21.2	21.6	22.3	19.4	
Older woman (aged 50+)	55.3	44.7		20.4	20.7	19.2	21.0	18.8	
Younger man (aged 18–49)	42.5	57.5		15.5	19.0	20.4	21.3	23.9	
Older man (aged 50+)	47.6	52.4		15.7	16.5	18.8	21.8	27.2	
Mean age of household head	58.4	59.4	n.s.	60.8	59.3	58.7	58.3	58.6	0.005
Main income earner			<0.0001						<0.0001
Younger woman (aged 18–49)	67.4	32.6		15.0	18.2	19.9	24.9	22.0	
Older woman (aged 50+)	54.7	45.3		19.6	21.5	18.8	20.6	19.5	
Younger man (aged 18–49)	41.9	58.1		16.6	18.7	20.1	19.9	24.6	
Older man (aged 50+)	46.8	53.2		15.0	16.5	19.0	22.3	27.2	
Mean age of main income earner	55.4	56.9	0.011	57.2	57.0	56.1	55.4	56.0	n.s.

§ Generations are calculated from the household roster: one=e.g. a married couple without children; two=e.g. parent/child or grandparent/child, etc.; three=e.g. grandparent/parent/child, etc.; skip-generation=e.g. grandparent/grandchild.

More than 60% of the single-person aged 50-plus households were among the two lowest income quintiles. In contrast, about 70% of the households with respondent and spouse were in the highest three income quintiles. Whilst the skip-generation was more common in the two lowest income quintiles (28.6 and 25.4%, respectively), the other types of generations were more frequent among the two highest income quintiles.

Household heads were classified into four groups by age and sex. The phenomenon of having a woman as head of household, either young or old, was more common in urban areas while men were more commonly heads of household in rural areas. The identical situation is seen for the household member identified as the main income earner.

Single person households were more common in the lower income quintiles (30.6%) while large household sizes were more common among the highest income quintiles (30.6%). In general, there was a significant relationship between household size and income quintile in which the household is located or assigned.

The category of the head of household, in terms of being young or old or being man or woman, was statistically related to the income quintile where older men as household head were wealthier (23.9% in 4th quintile and 27.2% in wealthiest (5th) quintile).

Similarly, male main income earners were wealthier (24.6% in 4th quintile and 27.2% in the 5th quintile). The mean age of the main income earner was higher for the households located in rural areas, but virtually the same across the income quintiles of the household.

The households were also assessed in terms of environmental risk factors, indoor air pollution, water, and sanitation. Table 5 details the characteristics of walls and floors, water, and sanitation. Overall, 89.1% of households had hard floors and the rest had earthen floors. In all the regions, hard floors were more common than the earthen ones except the Upper East and Upper West regions where earthen floors constituted 54.5 and 74.3%, respectively.

**Table 5:** Physical characteristics of households (%), by region and overall

Characteristics	All	Ashanti	Brong Ahafo	Central	Eastern	Greater Accra	Northern	Upper East	Upper West	Volta	Western
Floor											
Hard	89.1	97.7	93.3	95.0	90.4	93.9	83.0	45.5	25.7	81.3	94.5
Earth	10.9	2.3	6.7	5.0	9.6	6.1	17.0	54.5	74.3	18.7	5.5
Wall											
Cement	57.7	70.2	56.9	59.6	51.1	89.5	3.3	5.4	20.1	56.9	53.3
Mud	41.0	29.5	42.8	39.6	48.9	9.3	95.2	94.0	79.4	41.0	41.9
Plastic/metal	0.3	0.2	0.2	0.4	0	0.8	0.2	0.3	0	0.2	0.4
Thatched	0.9	0.1	0.1	0.4	0	0.4	1.3	0.3	0.5	1.9	4.4
Water											
Improved	84.9	93.1	90.7	70.4	88.1	93.7	66.8	81.0	98.2	29.6	83.0
Unimproved	15.1	6.9	9.3	29.6	11.9	6.3	33.2	19.0	1.8	70.4	17.0
Sanitation											
Improved	14.9	16.9	4.3	7.7	3.3	41.9	1.6	1.5	10.9	12.9	11.6
Unimproved	85.1	83.1	95.7	92.3	96.7	58.1	98.4	98.5	89.1	87.1	88.4

Improved drinking water: piped into household or yard; public standpipe, borehole, protected drug well, protected spring, rainwater collection and bottled water. Unimproved: unprotected dug well, unprotected spring, surface tank, tanker truck supplies.

Improved sanitation: connection to septic system, pour-flash latrine, covered dry latrine. Unimproved: uncovered dry latrine, bucket latrine, no facilities.

In all, 57.7% of households had cement walls, 41.0% had mud walls, and 0.9% had thatched walls. All the regions had cement walls as the most common feature of the dwellings of the households, except the three northern regions, where most of the walls of the households were of mud: 95.2% in Northern region, 94.0% in the Upper East region, and 79.4% in the Upper West region.

Eighty-five percent of households had improved sources of drinking water. All the regions had a higher percentage of households with improved sources of drinking water except the Volta region, where 70.4% of households had unimproved drinking water sources. The regions with the highest percentage of improved water source were the Upper West (98.2%), Greater Accra (93.7%), and Ashanti (93.1%).

Just 14.9% of households had improved sanitation, meaning 85.1% of all the households had unimproved sanitation. This picture was similar across all the regions, where unimproved sanitation ranged from a very high of 98.5% in the Upper East region to 58.1% in the Greater Accra region. Thus, even for the region with the lowest percentage of unimproved sanitation (Greater Accra) the figure was still above 50%.

## Discussion

SAGE Ghana Wave 1 generated valuable, reliable, and representative data on the household characteristics of ageing and older adults in Ghana. A number of methodological results were presented, along with some illustrative results from households. The study had a high response rate by household with some variation by region, lowest in the Northern urban strata and highest in the Eastern urban strata. Similarly, there was little evidence of age heaping with a Myers’ blended index of 11.9. An assessment of age reporting in the 2000 Census in Ghana resulted in a Myer's index of 15.3 (14).

### Comparison across regions

Three regions showed a percentage of people aged 65-plus higher than 10%, namely Central, Volta, and Eastern. Forty-one percent of the adult population were married, with three regions, Northern, Upper East, and Upper West, having more than half of their populations currently married while, consistent with the above-mentioned finding, Central, Eastern, and Volta regions report the highest percentages of widowed. The proportion widowed showed wide differences by sex; 15% of women were widowed compared to only 2.6% of men. Women formed 52.9% of household members, consistent with the national figure of 51.3% (8), highlighting the feminization of ageing.

In SAGE Wave 1, the urban population consisted of 51.1% of the population compared to 50.9% from the published 2010 Census estimates (8). Greater Accra is the wealthiest region with more than 89% of its population living in an urban setting.

The proportion with no formal education formed almost a quarter of the population and more than half had only primary school education. Significant regional differences were evident, especially in the Northern Regions where more than half of older adults had no formal education. The future implications of low education include poor access both to adequate income and health care for older adults. In a review by Boutayeb and Helmert (15) on human development, they concluded that social inequalities and health inequity existed between groups of a country despite improvements in the general literacy and per capita income of the countries of North Africa.

The total number of households 5,178 with a population of 27,270, giving a mean household size of 5.3 persons, somewhat higher than the 2010 Census estimates of 4.4 persons (8). This higher household size may reflect the differences seen in the composition of older households. Households with more than six members are more common in the Northern and in the Upper East region where the majority of the population live in rural areas.

Highly populated households were more present in the rural areas although those households were also the wealthiest (30.6% belonged to the highest income quintile). Single-member households present the worst situation, being poorer and older. In terms of single and multigenerational living arrangements, 174 households consisted of a married couple without children (one generation). The most common living arrangement, occurring in 2,290 households, was two-generation households consisting of parent/child or grandparent/grandchild. As Ghana's population continues to age, the expectation is that three-generational living arrangements, that is, older adults living with their children who have their own families, may become more common than it is currently. For older persons, this type of arrangement may provide social protection and enable them to bequeath their rich experience to their children and grandchildren; on the other hand the presence of older adults may impose extra financial and social cost on the family (4, 5). The social opportunities and challenges of the evolving living arrangements in Ghana will be worthy of consideration in national policy discussions.

### Characteristics of the household across regions

The wealthiest Greater Accra region is also reflected by household characteristics like very high percentages of hard floor, cement wall, and improved drinking source. The Upper East, Upper West, and Northern regions illustrate variations in dwelling characteristics in the three poorest regions. Only 3.3% of the Northern region households had cement walls versus 20.1% among the Upper West region dwellings. Improved sanitation ranges between 1.5% in the Upper East region and 10.9% in the Upper West region.

Despite Greater Accra being comparatively wealthier, just 42% of households in this region had improved sanitation. The Ashanti region, the second wealthiest region, provides improved sanitation to only a 17% of its population. Ten percent of households in GLSS 5 and 15.4% in the 2010 Census had access to a flush toilet. The pan/bucket latrine, which is actively being discouraged and phased out because of its minimum sanitary standards, forms the smallest proportion (20%), and about 19% of the population has no toilet facility in their homes, while in the three northern regions, up to 72% of households have no access to any form of toilet facility according to the 2010 Census (8). High rates of unimproved sanitation were found throughout the country in SAGE. This poses a serious threat to healthy living as the traditional type of sanitary facilities mostly contributes to unhygienic environment and facilitates easy transmission of diseases (16).

Risk pooling for financial accessibility to health services was low among older household members in SAGE Ghana (2007/08), almost three quarters did not have insurance coverage, despite Ghana introducing the National Health Insurance Scheme (NHIS) by an Act of Parliament Act 650 in August 2003 (17). As of December 2010, over 18 million Ghanaians (over 70% of entire national population) had subscribed to the scheme, out of which over 8 million, representing 34% of the population, were active card bearers. The high patronage attests to the fact that Ghanaians have embraced the NHIS as the preferred health care financing mechanism (18). The challenge for the older population in Ghana is that the retirement age for public sector workers is 60 years; while the NHIS provide free care to those aged 70-plus years (17). A policy to provide health insurance for those adults aged 61–69 years is worth pursuing and may need to be incorporated in the Draft National Policy on Ageing.

While the results of this study are subject to the limitations of self-report, much of the subject matter presented in this article was also largely observable by the survey team. Interviewers were given the opportunity to rate the reliability of the household respondents, and note any corrections based on objective observations. As well, the focus of SAGE being older households, the comparisons to other published results are similar, but not strictly comparable without access to the microdata.

## Conclusion

SAGE Wave 1 provides data to describe the household characteristics of older adults in Ghana using approaches similar to other large data collection efforts. SAGE uses sound research methodology in a nationally representative sample to inform policy and planning for the older population in Ghana. In this study, significant differences were observed in households across regions, especially regions in the Northern part of the country. A comprehensive national policy on ageing is crucially important to ensure healthy living arrangements and households as elements to maintain health and well-being in ageing populations across all parts of Ghana – SAGE can provide the data needed to inform this policy.

## Appendix 1

**Table:** Questionnaire types and description of contents, SAGE Wave 1

Questionnaire type	Domain	Wave 1 measures
Household	Household identification, contact and sampling details	Identification and contact details; structure of household; dwelling characteristics; improved water, sanitation and cooking facilities
	Transfers and support networks	Family, community and government assistance into and out of the household; informal personal care provision/receipt
	Assets, income and expenditure	List of household assets; sources and amount of household income; improved household expenditure on food, goods and services, health care
	Household care and health insurance	Persons in household needing care; mandatory and voluntary health insurance coverage
Individual	Sociodemographic characteristics	Sex; age; marital status; education; ethnicity/background; religion; language spoken; area of residence; employment and education of parents; childhood residence, migration
	Work history and benefits	Length of time worked; reasons for not working; type of employment; mode of payment; hours worked; retirement
	Health states and descriptions	Overall self-rated health; eight self-rated health domains (affect, mobility, sleep/energy, cognition, interpersonal activities, vision, self-care and pain); 12-item WHO Disability Assessment Schedule, Version 2 (WHODAS-II); activities of daily living (ADLs); instrumental activities of daily living (IADLs); vignettes on health state descriptions
	Anthropometrics, performance tests and biomarkers	Measured blood pressure; self-report and measured height and weight; measured waist and hip circumference; timed walk; near- and distant vision tests; grip strength, executive functioning (verbal recall, digit span forwards and backwards, verbal fluency); spirometry; non-fasting fingerprick blood sample (stored at -20C) as dried blood spots
	Risk factors and preventive health behaviours	Smoking; alcohol consumption; fruit and vegetable intake; physical activity (GPAQ)
	Chronic conditions and health services coverage	Self-reported and symptomatic reporting of arthritis; stroke; angina (Rose Questionnaire); asthma; and, depression (ICD-10, DSM-IV). Self-reporting of diabetes; chronic lung disease; hypertension; cataracts; oral health (edentulism); injuries; cervical and breast cancer screening
	Health care utilization	Past need for health care; reasons for health care or for not receiving health care; inpatient and outpatient health care: number of admissions/visits within the past 3 years (inpatient) or 1 year (outpatient); reasons for admission/visit; details of hospital or provider; costs of hospitalization or health care visit; satisfaction with treatment; health system responsiveness; vignettes for responsiveness of health services
	Social cohesion	Community involvement and social networks; perceptions of other people and institutions; safety in local area; stress; interest in politics and perceptions of government
	Subjective well-being and quality of life	Perceptions about quality of life and well-being; 8-item WHO Quality of Life measure (WHOQoL); Day Reconstruction Method (DRM)
	Impact of caregiving	Household members needing care; type of care required; length of time spent on care; costs of care; impact of providing care on career well-being
Proxy	IQ Code	IQ Code;
	Health state descriptions	All measures described above for individual data
	Chronic conditions	All measures described above for individual data
	Health care utilization	All measures described above for individual data
Proxy validation	Quality control measure	Supervisor checks that proxy interview warranted and completed
Retest	Quality control measure	Selected key variables for household and individual questionnaires repeated up to one week after initial interview.
Mortality (verbal autopsy)	Deaths and cause of death	Verbal Autopsy for all deaths within past 24 months in households

Note: Section 9000 of the individual questionnaire allowed the interviewer to document observations during the interviews.
